# Different Land Use Systems in the Brazilian Cerrado and Their Effects on Soil Bacterial Communities

**DOI:** 10.3390/microorganisms13040804

**Published:** 2025-04-01

**Authors:** Jefferson Brendon Almeida dos Reis, Thayssa Monize Rosa de Oliveira, Maria Regina Silveira Sartori da Silva, Fabyano Alvares Cardoso Lopes, Alessandra Monteiro de Paula, Nadson de Carvalho Pontes, Helson Mario Martins do Vale

**Affiliations:** 1 University of Brasilia, Institute of Biological Sciences, Brasília 70910-900, DF, Brazil; jeffersonalmeidareis@gmail.com (J.B.A.d.R.); rsartori@unb.br (M.R.S.S.d.S.); 2 Centro de Excelência em Bioinsumos (CEBIO), Instituto Federal Goiano, Campus Morrinhos, Morrinhos 75650-000, GO, Brazil; thayssa28@hotmail.com (T.M.R.d.O.); nadson.pontes@ifgoiano.edu.br (N.d.C.P.); 3 Laboratory of Microbiology, Federal University of Tocantins, Porto Nacional 77500-000, TO, Brazil; flopes@mail.uft.edu.br; 4 University of Brasilia, Faculty of Agronomy and Veterinary Medicine, Brasília 70910-900, DF, Brazil; alessandramp@unb.br

**Keywords:** bacteria community, cover crop, regenerative agriculture, soil carbon, soil enzymes, zinc

## Abstract

The effect of agricultural practices on soil bacterial communities is not constant and depends a lot on the climatic context, changes in the soil characteristics, land use, and agricultural strategy. Thus, knowledge about how different land use systems in the Cerrado influence the diversity and taxonomic structure of microbial communities under the same soil type remains limited. In this context, the objective of this work was to analyze and compare the bacterial communities of Cerrado soil under two different land use systems (cover crop and potato cultivation) and in a neighboring native Cerrado area. For this, we used high-throughput amplicon sequencing of 16S rRNA genes (metabarcoding) to characterize the bacterial community at different taxonomic levels in a native Cerrado area, in a potato crop area, and in an area with cover crops. Our data indicated significant impacts on soil physicochemical properties and enzymatic activity, which directly reflect the dynamics of bacterial communities. The three bacterial phyla with the highest relative abundance in the three areas were Proteobacteria, Actinobacteriota, and Acidobacteriota. At the taxonomic class level, small variations were observed among areas, while at the amplicon sequence variant (ASV) level, these variations were more pronounced. The alpha diversity indices showed that the bacterial communities among the areas are rich and diverse. Bray–Curtis and Jaccard distance-based PCoA demonstrated an overlap of bacterial communities present in the cover crop area with the native Cerrado area and separation from the potato cultivation area. The in silico prediction demonstrated that the native Cerrado area presented the highest values of functional diversity of the soil bacterial community compared to the others. Thus, our results provide a holistic view of how different land use systems in the Cerrado can influence the taxonomic and functional diversity of soil bacterial communities.

## 1. Introduction

Soils are a highly functional environment, essential for the stability of terrestrial ecosystems, influencing and boosting the flow of energy, nutrient cycling, and environmental quality, and constitute one of the largest reservoirs of microbial diversity in the global context [1,2,3]. In these environments, bacteria are the most abundant microbial group and play a myriad of roles in biogeochemical cycles, nutrient cycling, and energy flux [4,5]. Thus, these microbial communities are key players in numerous ecosystem services in the soil environment [4,5].

The taxonomic structure of bacterial communities and their biological multifunctionality can be influenced by land use as a result of changes in the physical, chemical, and biological characteristics of the soil [6,7,8,9]. In this context, different types of agricultural practices (e.g., crop rotation, soil preparation, irrigation, fertilization) can positively or negatively influence the biodiversity and functionality of bacterial communities [6,10]. Soils managed organically or with agricultural practices that employ rotational systems with cover crops have higher levels of organic matter and a greater abundance of microbial activity [6,11]. On the other hand, conventional intensive agriculture can cause a loss of soil microbial biodiversity and a reduction in its functionality [12]. However, the effect of agricultural practices on soil bacterial communities is not constant and depends a lot on the climatic context, soil characteristics, and agricultural strategy [13].

In tropical regions, agricultural expansion is among the main threats to biodiversity and soil stability [14]. For example, it is estimated that since 1960, approximately half of the entire area of the Brazilian Cerrado (neotropical savanna) has been domesticated for agriculture, placing this important biome on the list of critical areas for biodiversity conservation worldwide [15,16,17,18]. From an economic point of view, the benefits of commercial agriculture in the Cerrado are a success, so the region of the biome is the main grain producer in the country [19]. However, changes in land use in the Cerrado have led to landscape fragmentation, loss of biodiversity, biological encroachment, and changes in the water and carbon cycles [16,17,18,19].

Despite the ecological and economic importance of the Cerrado, there are still few studies carried out to understand how the agricultural practices employed in this biome drive changes in the taxonomic structure and functionality of soil bacterial communities [20,21,22]. Thus, knowledge about how different land use systems in the Cerrado influence the diversity and taxonomic structure of microbial communities under the same soil type remains limited. The characterization of the bacterial diversity and aspects that inform soil health can provide a better and more comprehensive understanding of the system, propose more sustainable land use practices, and contribute to improvements in environmental benefits. In this context, the objective of this work was to analyze and compare the bacterial communities of Cerrado soil under two different land use systems (cover crop and potato cultivation) and in a neighboring native Cerrado area. We hypothesize that (i) agricultural land use systems influence soil physicochemical properties, and (ii) bacterial communities differ between these systems, showing distinct taxonomic and functional profiles in areas with cover crops, potato cultivation, and native Cerrado soil. To test this hypothesis, we used high-throughput amplicon sequencing of 16S rRNA genes (metabarcoding) to characterize the bacterial community at different taxonomic levels in a native Cerrado area (Cerrado *sensu stricto*), in a potato growing area, and in an area with cover crops. Furthermore, we used molecular network analyses to find taxa that co-occur between different areas; we analyzed the functional profile of the main microbial groups found, and we evaluated the enzymatic profile and the physical and chemical characteristics of the soil among the three areas. Thus, our results provide a holistic view of how different land use systems in the Cerrado can influence the taxonomic and functional diversity of soil bacterial communities.

## 2. Materials and Methods

### 2.1. General Description of the Areas and Soil Sampling

Three areas were selected for evaluation within the same agricultural property in the municipality of Cristalina, Goias, Brazil. The climate type is Aw, according to Koppen’s classification. The Cerrado is a tropical wet–dry savanna climate marked by two distinct seasons: rainy (October–April) and dry (May–September). To reference the original soil bacteria community, the first area (geographic coordinates: 16°11′00.5″ S 47°27′33.9″ W) consisted of a native Cerrado (NC) area (Cerrado *sensu stricto*), an adjacent area with a native forest forming part of the property’s legal reserve and neighboring the cultivation areas. The second area (geographic coordinates: 16°10′58.5″ S 47°27′46.4″ W) was occupied by a joint cover crop (CC) of cereal rye (*Secale cereale*) and buckwheat (*Fagopyrum esculentum*) (45 days old). These cover crops were intended to recover the area after a potato crop. The third area (geographic coordinates: 16°01′22.1″ S 47°24′54.0″ W) had a commercial potato crop (PC) already in the harvest phase. The areas where cover crops and potatoes were grown were irrigated with a sprinkler irrigation system via a central pivot.

Soil samples were collected in three areas at the end of a dry winter (September 2022). Sampling was performed as described by Moreira et al. [23]. For soil sampling, the main point was georeferenced and sampled 12 points, forming two concentric circles around the sample point with a distance of 3 m; the outer circle had a radius of 6 m, resulting in a composite soil sample. Samples were collected in three main points (0–0.10 m depth), resulting in three composite soil samples per area. Soil samples were placed in plastic bags and stored at 4 °C to be later analyzed.

### 2.2. Physicochemical and Soil Enzyme Analysis

The soil was classified as a Red-Yellow Latosol class, according to the Brazilian taxonomy system Embrapa [24], corresponding to Oxisols Ustox, according to the U.S. soil taxonomy system [25]. For the chemical characterization, soil samples were analyzed in triplicate for pH (H_2_O, 1:5); extractable Al (determined through titration with 0.025 mol L^−1^ NaOH); Ca and Mg (extracted with 1 mol L^−1^ KCl and determined through atomic absorption); extractable P (extracted with an acid solution of 0.0125 mol L^−1^ sulfuric acid and 0.05 M hydrochloric acid–Mehlich-1, determined through the blue-Mo method); K (Mehlich-1, determined through flame spectrophotometry); C (Walkely–Black method); Zn (Mehlich-1, determined through atomic absorption); titratable acidity (H + Al) extracted with 0.5 mol L^−1^ calcium acetate buffer pH 7.0 and determined by volumetric analysis using 0.025 mol L^−1^ NaOH in the presence of phenolphthalein as an acid–base indicator; and effective cation exchange capacity (CEC—the sum of all base cations plus exchangeable Al and H). These measurements followed the procedure as described by Silva [26]. The soil moisture was determined after drying the samples overnight at 105 °C. The activities of β-Glucosidase, arylsulfatase, and phosphatase (related to the carbon, sulfur, and phosphorus cycle, respectively) were assayed at their optimal pH values (6.0, 6.5, and 5.8, respectively) in triplicates, including one control, as described in Tabatabai [27].

### 2.3. Amplicon Sequencing

Environmental DNA (eDNA) from the soils was extracted using the FastDNAR SPINS kit (MP Biomedical, Santa Ana, CA, USA) according to the manufacturer’s instructions. The V3-V4 target sequence was accessed by the primer pair 341F (5′-CCTAYGGGRBGCASCAG-′3) and 806R (5′-GGACTACNNGGGTATCTAAT′-3). Sequencing was performed on a MiSeq platform (Illumina, San Diego, CA, USA). Quality analysis of sequences was performed using BBDuk version 38.34 (BBMap–Bushnell B.–sourceforge.net/projects/bbmap/) with the following parameters: removal of Illumina adapters, including Illumina artifacts and the PhiX Control v3 Library; ktrim = l; k = 23; mink = 11; hdist = 1; minlen = 50; --tpe; --tbo; qtrim = rl; trimq = 20; ftm = 5; maq = 20. The output sequences were imported into QIIME2 version 2022.11 for bioinformatics analyses [28]. The qiime2-dada2 plugin is a complete pipeline that was used for filtering, dereplication, turning paired-end fastq files into merged, and removing chimeras [29]. Taxonomic assignments were determined for amplicon sequence variants (ASVs) using the QIIME2 feature classifier [30] classify-sklearn against SILVA 138 Ref NR 99 [31]; the database was trained with the Naive Bayes classifier. The taxonomic assignments for the ASVs were made at different taxonomic levels, ranging from phylum to species. The specific level of classification depends on the similarity of the ASV to sequences deposited in the SILVA database.

### 2.4. Statistical Analyses

Statistical and diversity analyses were performed using R language scripts (version 4.1.3). The annotation of the taxonomic sharing network was performed using the CYTOSCAPE software version 3.9.1 [32]. For this, the ASV table at phylum/class/order/family/genus level generated from QIIME 2 was used as input.

Relative abundance at different taxonomic levels was calculated in the “Phyloseq” package (version 1.48.0) [33]. We used the “pheatmap” package (version 1.0.12) to visualize the most abundant ASV between areas. Metrics of richness (S), Shannon (H’), Simpson’s index 1, and Pielou evenness (J) were calculated using the R software package using the “vegan” package (version 2.6.8) [34]. The dabestr package (version 2023.09.12) [35] was used to generate estimation graphs in the alpha diversity analyses. We chose the method of estimating effect sizes and their uncertainty, allowing the visualization of complete sampling curves and effect sizes in order to highlight the variability in the alpha diversity data. The effect strength values, accompanied by the 95% confidence intervals, were represented in Gardner–Altman plots for all alpha diversity metrics, highlighting those whose confidence intervals do not overlap zero (5000 bootstrap samples). Principal coordinate analysis (PCoA) and two-way permutational multivariate analysis of variance (PERMANOVA) were used to compare the beta diversity of bacterial communities between samples based on the Bray–Curtis and Jaccard distance matrix using the “vegan” package (version 2.6.8) [34]. To calculate the Bray Curtis distance, the community data matrix was transformed by Hellinger, and for the Jaccard distance, the community data matrix was transformed into binary data (0 or 1) using the “vegan” package.

We estimated the decline of zeta diversity (ζ) with the “zetadiv” package (version 1.2.1) [36]. Briefly, zeta diversity (ζ) can be defined as the average number of ASVs shared across n number of sites or sampling units; the number of sites or sampling units that are used to estimate zeta diversity is referred to as zeta order (hereafter ζ i for i different number of sites or sampling units) [37]. For zeta order 1, zeta diversity corresponds to the average richness of ASVs per location or sampling unit (ζ 1), while for order 2, it is the average number of ASVs shared across two locations or sampling units (ζ 2) [37]. We calculated zeta decay and retention rate. Zeta decline represents a decrease in the average number of shared ASVs with increasing n of sites or sampling units (=zeta order). The slope of the zeta decay indicates whether the compositional change is mainly due to rare (homogeneous decay) or common (heterogeneous decay) ASVs. The retention rate (zeta ratio ζ i/ζ i + 1) quantifies the proportion of ASVs that are retained with the addition of more sites or sampling units to estimate zeta diversity. The decline of zeta diversity is generally described by the power law or exponential function and is informative about the underlying processes that drive the differentiation of ASV composition and the role of common and rare ASVs in ASV turnover patterns [38]. Power law decline suggests that ASVs have a unique chance of occurring in a location, indicating that ecological niche-based processes drive species turnover. Exponential decline indicates that ASVs have an equal chance of occurring in a location [38].

Network analysis was performed to visualize community structure and identify the co-occurrence patterns of soil bacteria ASVs among the three treatments. Co-occurrence network correlations were calculated using an integrated network analysis pipeline (iNAP) [39]. To ensure statistical robustness, only ASVs with more than 100 reads and occurring in at least half of the samples were considered. The SparCC (Sparse Correlations for Compositional) method was used to calculate the network with the following parameters: correlation strength exclusion limit = 0.1, 100 times shuffled, threshold value > 0.6, and *p*-value = 0.001. The network was visualized using CYTOSCAPE version 3.9.1. The structural attributes of the networks were explored using the “Networking Analyze” tool [40]. We use MCODE (version 2.0.3) to explore the loosely connected nodes [41].

In addition, we apply the multinomial species classification method (CLAMtest) with the “clamtest” function of the “vegan” package to understand the ASVs that show a preference for the area. We used a coverage threshold of 10, an alpha of 0.005, and a specialization value of 0.66, which is considered conservative [42]. CLAMtest uses a multinomial model based on estimating the relative abundance of species in two habitats, classifying species into one of four groups: (1) generalists; (2) specialists in area X (cover crop; native Cerrado; potato crop); (3) area Y specialists (cover crop; native Cerrado; potato crop); and (4) very rare species.

The functional profiles of the soil bacterial community present in the three areas were predicted in silico using the “Global mapper” module of the ’iVikodak’ platform [43]. For this, the abundance data of ASVs at the taxonomic level of order were provided to the “Global mapper” module, and the functional prediction was based on KEGG. Additionally, we employed the R package microeco (version 1.9.1) to assess the functional specialization of the bacterial community using the FAPROTAX database (version 1.2.9). The functional specialization was calculated with the cal_spe_func(prok_database = “FAPROTAX”) method, and the abundance-weighted functional specialization was determined using cal_spe_func_perc(abundance_weighted = TRUE).

Pearson’s correlation coefficient was used to determine the correlation between measured variables. We use the “corrplot” package to visualize the heatmap with the correlation values. Data on environmental variables were also analyzed using the Kruskal–Wallis statistical test (*p* < 0.05). The data on environmental variables were analyzed using the method of estimating effect sizes and their uncertainty, which allowed the visualization of complete sampling curves and effect sizes to highlight the variability in the data [35]. The resulting effect size values and 95% confidence intervals were represented in Gardner–Altman plots, with a focus on those intervals that do not overlap zero (5000 bootstrap samples). Principal component analysis (PCA) was used to describe the patterns of variation explained by soil parameters using the “FactoMineR” (version 2.11) and “ggplot2” (version 3.5.1) packages.

## 3. Results

### 3.1. Physicochemical and Soil Enzyme Analysis

**Figure 1:**
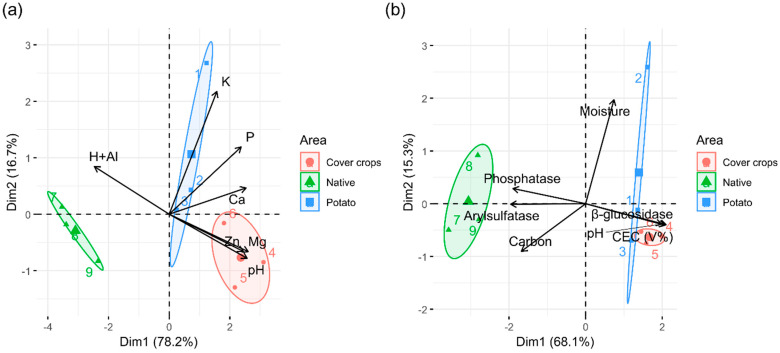
Principal component analysis (PCA) to detect the correlation between physical–chemical variables and soil enzymes among cover crop area, native Cerrado, and potato cultivation. (**a**) PCA considering micro-macronutrient contents and pH; (**b**) PCA considering soil enzymes, carbon, moisture, and pH. Dim 1 (PC1) and Dim 2 (PC2) represent the x and y axes, respectively. Ellipses show replicates per area. Each color corresponds to an area. The length of the arrows represents the strength of association of the respective soil properties with the respective areas. The angle between the vectors indicates the degree of their relationship (smaller angle means high correlation). Al: Aluminum; Ca: Calcium; K: Potassium; Mg: Magnesium; P: Phosphorus; Zn: Zinc; Figure created by R software version 4.1.1. using the “ggplot2” package.

**Figure A1:**
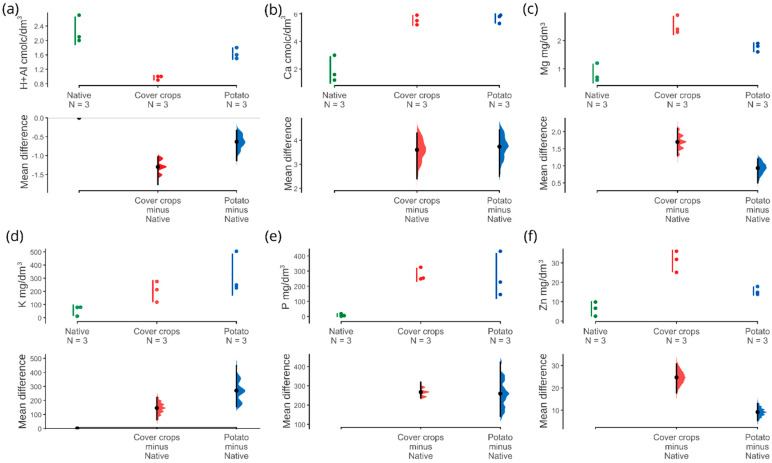
Gardner–Altman plots representing soil nutrient and cation values in areas of cover crop (CC), potato crop (PC), and native Cerrado (NC) (Cerrado sensu stricto). Variations in nutrient and cation concentrations were analyzed among the different areas. The plots present the strengths of effect (Hedge’s g) and their 95% confidence intervals for nutrients and cations where the confidence intervals do not include the value 0, indicating significant differences. In Gardner–Altman plots, the distribution of sampled values is displayed at the top, while Hedge’s g is shown at the bottom as a dot, accompanied by a horizontal line on the axis. The vertical error bars represent the 95% bootstrap confidence intervals, based on 5000 bootstrap samples, together with the distribution of the resamplings. Abbreviations: H + Al: aluminum; Ca: calcium; K: potassium; Mg: magnesium; P: phosphorus; Zn: zinc. Concentratio of aluminum (**a**), calcium (**b**), magnesium (**c**), potassium (**d**), phosphorus (**e**), zinc (**f**).

**Figure A2:**
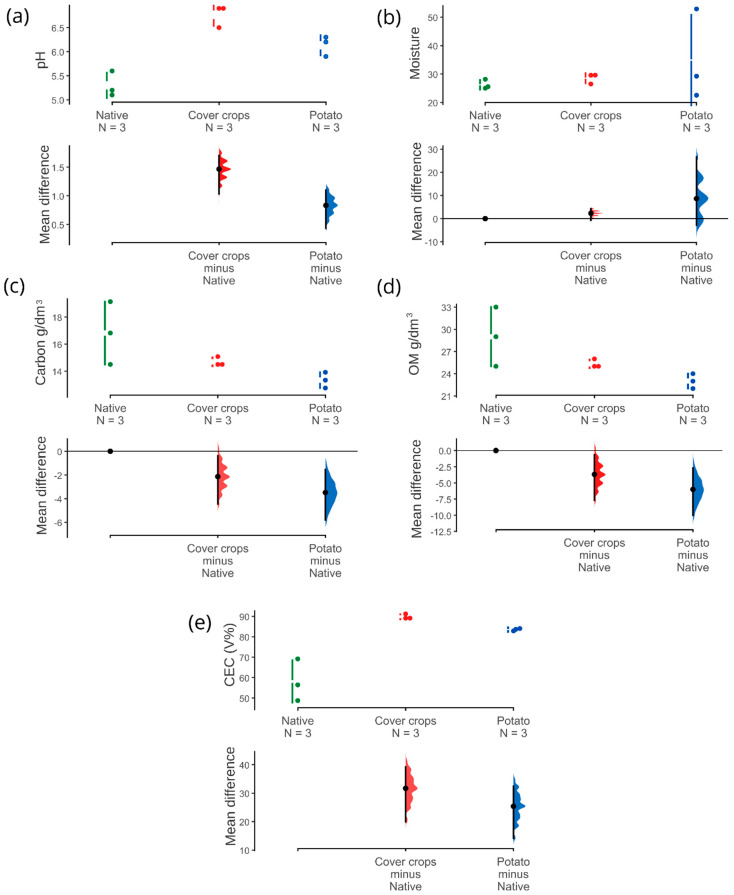
Gardner–Altman plots representing soil pH, cation exchange capacity, organic matter, and carbon values in areas of cover crop (CC), potato cultivation (PC), and native Cerrado (NC) (Cerrado sensu stricto). The observed variations in pH (**a**), moisture (**b**), carbon (**c**), organic matter (**d**), and cation exchange capacity (**e**) were analyzed among the different areas. The plots present the effect strengths (Hedge’s g) and their 95% confidence intervals for nutrients and cations where the confidence intervals do not include the value 0, indicating significant differences. In the Gardner–Altman plots, the distribution of sampled values is displayed at the top, while Hedge’s g is shown at the bottom as a dot, accompanied by a horizontal line on the axis. The vertical error bars represent the 95% bootstrap confidence intervals, based on 5000 bootstrap samples, along with the distribution of the resamplings. OM: organic matter; CEC (V%): cation exchange capacity.

**Figure A3:**
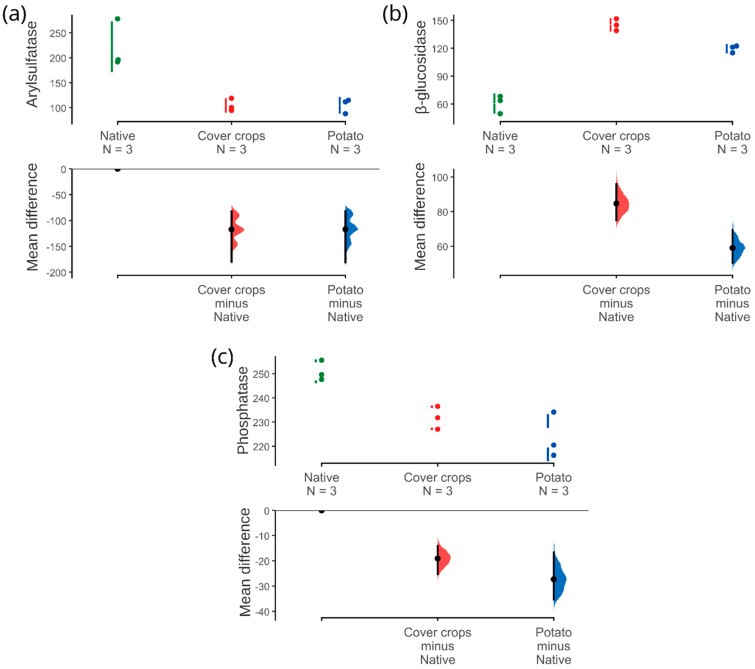
Gardner–Altman plots representing soil enzyme values in areas of cover crop (CC), potato crop (PC), and native Cerrado (NC) (Cerrado sensu stricto). The observed variations for soil enzymes arylsulfatase (**a**) β-glusosidade (**b**) and phosphatase (**c**) were analyzed among the different areas. The plots present the effect strengths (Hedge’s g) and their 95% confidence intervals for nutrients and cations where the confidence intervals do not include the value 0, indicating significant differences. In the Gardner–Altman plots, the distribution of sampled values is displayed at the top, while Hedge’s g is shown at the bottom as a dot, accompanied by a horizontal line on the axis. The vertical error bars represent the 95% bootstrap confidence intervals, based on 5000 bootstrap samples, together with the distribution of the resamplings. OM: organic matter; CEC (V%): cation exchange capacity.

The results of micro and macro-nutrient content, cation exchange capacity (CEC), humidity, carbon, organic matter, and enzymes among the three areas are presented in Appendix A (Figure A1, Figure A2 and Figure A3). Bootstrap analysis revealed significant differences in the levels of calcium (Ca), aluminum (Al), phosphorus (P), potassium (K), magnesium (Mg), zinc (Zn), carbon, organic matter (OM), pH, cation exchange capacity (CEC), and soil enzymatic activities (arylsulfatase, phosphatase, and β-Glucosidase). Based on soil factors, the Principal component analysis (PCA) demonstrated a clear separation of the areas with no overlap (Figure 1a,b). Samples from the potato area were distributed across two quadrants, indicating greater environmental heterogeneity compared to the other two areas. The PCA results for micro and macronutrients revealed that the Dim1-2 components explained 94.9% of the variance (Dim1: 78.2% and Dim2: 16.7%) (Figure 1a). When PCA was applied to enzyme activity, pH, and carbon indices, the Dim1-2 components accounted for 83.4% of the variance (Dim1: 68.1% and Dim2: 15.3%) (Figure 1b). Furthermore, the native Cerrado (NC) area was correlated with higher levels of aluminum (Al), carbon, arylsulfatase, and phosphatase. The cover crop (CC) area was correlated with higher zinc (Zn) levels, higher pH, and β-Glucosidase values. The area cultivated with potatoes was correlated with higher potassium (K) and calcium (Ca) levels and higher humidity.

**Figure 2:**
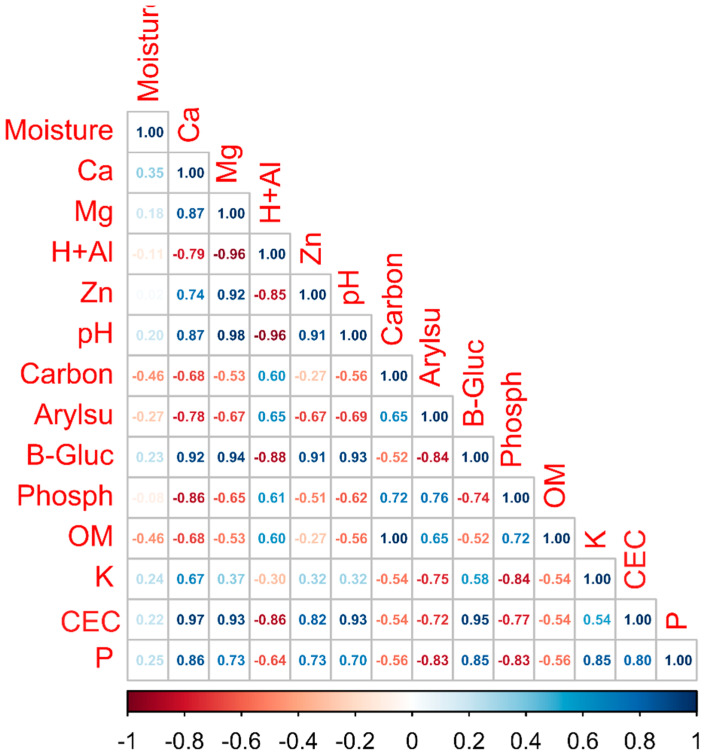
Pearson correlation heatmap between soil physicochemical characteristics and soil enzyme activity among cover crop areas, native Cerrado, and potato cultivation areas. The circles show the intensity of the correlation: the larger the circle, the greater the strength of the correlation. Colors show the nature of the correlations: positive correlations are shown in blue, and negative correlations are in red. H + Al: aluminum; Ca: calcium; K: potassium; Mg: magnesium; P: phosphorus; Zn: zinc; OM: organic matter; CEC: cation exchange capacity; B-Gluc: B-Glucosidase; Arylsul: arylsulfatase; Phosph: phosphatase.

**Figure A4:**
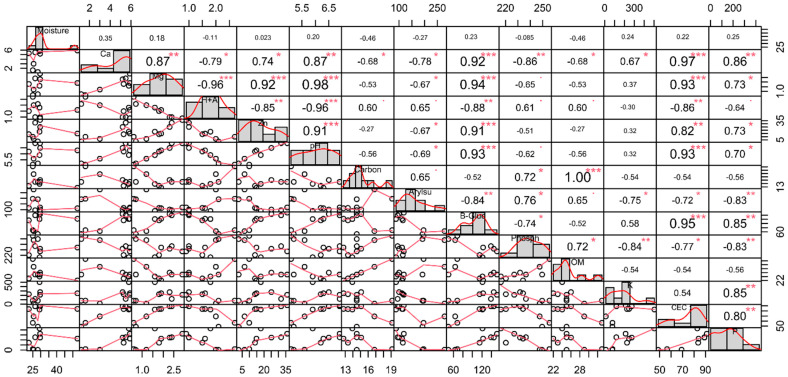
More details about Pearson correlation heatmap between soil physicochemical characteristics and soil enzyme activity among cover crop areas, native Cerrado, and potato cultivation areas. H + Al: aluminum; Ca: calcium; K: potassium; Mg: magnesium; P: phosphorus; Zn: zinc; OM: organic matter; CEC: cation exchange capacity; B-Gluc: B-Glucosidase; Arylsul: arylsulfatase; Phosph: phosphatase. *: *p* < 0.05; **: *p* = 0.01. ***: *p* < 0.001.

We performed a Pearson correlation analysis of the physicochemical variables and enzyme activity of the soil (Figure 2) and Appendix A (Figure A4). Arylsulfatase enzyme activity was significantly correlated with Ca (Pearson correlation = −0. 78), Mg (Pearson correlation = −0.67), Zn (Pearson correlation = −0.67), and pH (Pearson correlation = −0.69). Phosphatase activity was significantly correlated with the contents of Ca (Pearson correlation = −0.86), carbon (Pearson correlation = 0.72), arylsulfatase (Pearson correlation = 0.76), and β-Glucosidase (Pearson correlation = −0.74). β-Glucosidase activity correlated with Ca (Pearson correlation = 0.92), Mg (Pearson correlation = 0.94), Al (Pearson correlation = −0.88), Zn (Pearson correlation = 0.91), pH (Pearson correlation = 0.93), and arylsulfatase (Pearson correlation = −0.84).

### 3.2. Relative Abundance

**Figure 3:**
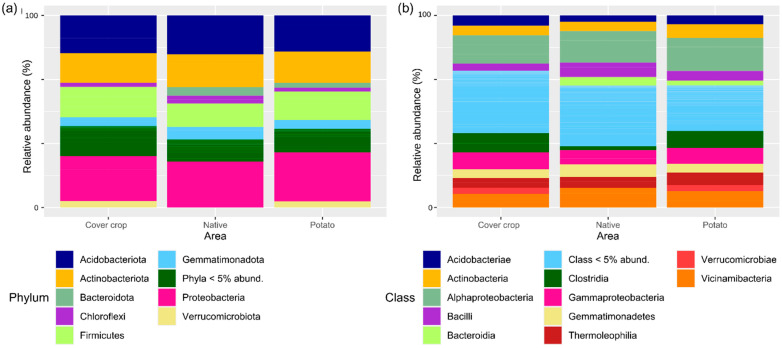
Relative abundance of the soil bacterial community in a cover crops area, native Cerrado, and potato cultivation. (**a**) Relative abundance at the phylum (**a**) and class (**b**) level.

Illumina MiSeq high-throughput sequencing analysis of the 16S rDNA gene was conducted to determine the bacterial community structure. The readings obtained from different samples ranged from 100,835 to 119,130 per sample. These sequences were assigned to 1730 ASVs. These ASVs have been classified into 37 phyla, 79 classes, more than 100 orders, families, and genera. The main bacterial taxa (mean relative abundance > 10%) belonged to the phylum Proteobacteria, Acidobacteriota, Actinobacteriota, and Firmicutes (Figure 3a). The Proteobacteria phylum is the most abundant in the three areas, with an average abundance of 25.29% ± 1.20% in the PC area, 23.85% ± 2.41% in the NC area, and 23.64% ± 2.37% in the CC area. The phyla Bacteroidota, Chloroflexi, and Gemmatimonadota showed an average relative abundance of >5% in the three areas. Verrucomicrobiota presented an average relative abundance greater than >5% only in the PC and CC areas. The other phyla had an average relative abundance of <5%. The main classes found were Alphaproteobacteria, Acidobacteria, Actinobacteria, Bacilli, Bacteroidia, Clostridia, Gammaproteobacteria, Gemmatimonadetes, Thermoleophilia, Verrucomicrobiae, and Vicinamibacteria (average abundance > 5%) (Figure 3b). Alphaproteobacteria was the dominant class in the three areas, with an average abundance of 16.55% ± 0.35% in the PC area, 16.14% ± 3.27% in the NC area, and 14.55% ± 0.53% in the CC area. Verrucomicrobiae presented an average relative abundance of >5% in the CC and PC areas, while Bacteroidia presented an average abundance of >5% in the NC and PC areas. The Clostridia class showed increased abundance in the CC (10.59% ± 4.50%) and PC (9.21% ± 2.44) areas compared to the NC area (5.05% ± 1.66%).

**Figure 4:**
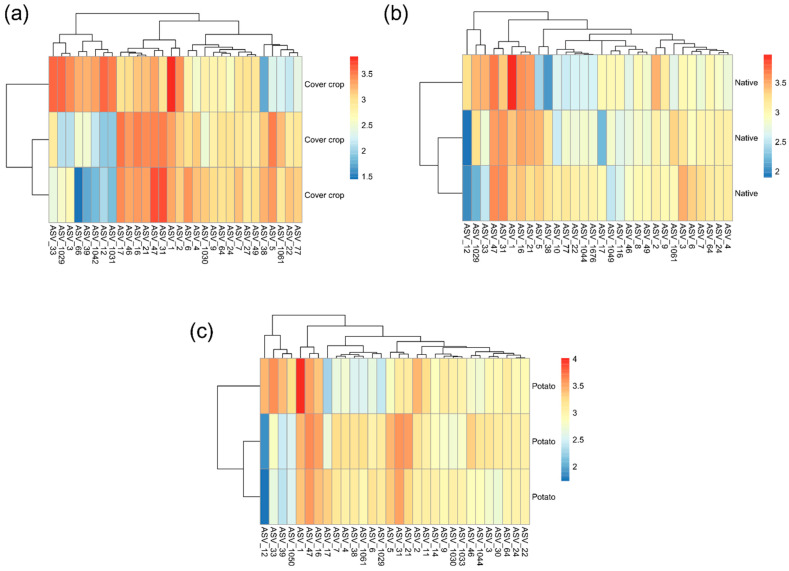
Heatmap representing the relative abundance of the 30 most abundant ASVs in the native Cerrado cover crop and potato cultivation areas. (**a**) Cover crops; (**b**) native; (**c**) potato. The lines are the sampling effort (*n* = 3). The columns show the 30 most abundant ASVs. The colors represent the relative abundance of each ASV: the redder the bar, the greater the abundance; the bluer it is, the lower the abundance. To facilitate data visualization, the number of reads, which is equivalent to abundance, was represented on a logarithmic scale + 1. The dendrograms show the grouping of ASVs (columns) and samples (rows) based on Euclidean distance. The figures were plotted using the “pheatmap” package implemented by the R software version 4.3.0.

The 30 most abundant ASVs in each of the three areas are shown in Figure 4. ASV_1 (Xanthobacteraceae), ASV_47 (Vicinamibacterales), and ASV_31 (Vicinamibacteraceae) were the most abundant in the three areas. In the CC area, the fourth most abundant ASV was ASV_17 (Lachnospiraceae), followed by ASV_16 (Gemmatimonadaceae), ASV_21 (Pyrinomonadaceae), ASV_5 (Sphingomonas), ASV_46 (Lachnospiraceae), and ASV_2 (Bradyrhizobium). In the NC area, they were ASV_16 (Gemmatimonadaceae), ASV_21 (Pyrinomonadaceae), ASV_3 (Lactobacillus), ASV_2 (Bradyrhizobium), ASV_5 (Sphingomonas), and ASV_1029 (Turicibacter). In the PC area, they were ASV_21 (Pyrinomonadaceae), ASV_5 (Sphingomonas), ASV_2 (Bradyrhizobium), ASV_33 (Acidobacteriales), and ASV_11 (Bifidobacterium).

### 3.3. Alpha, Beta, and Zeta Diversity

**Figure A5:**
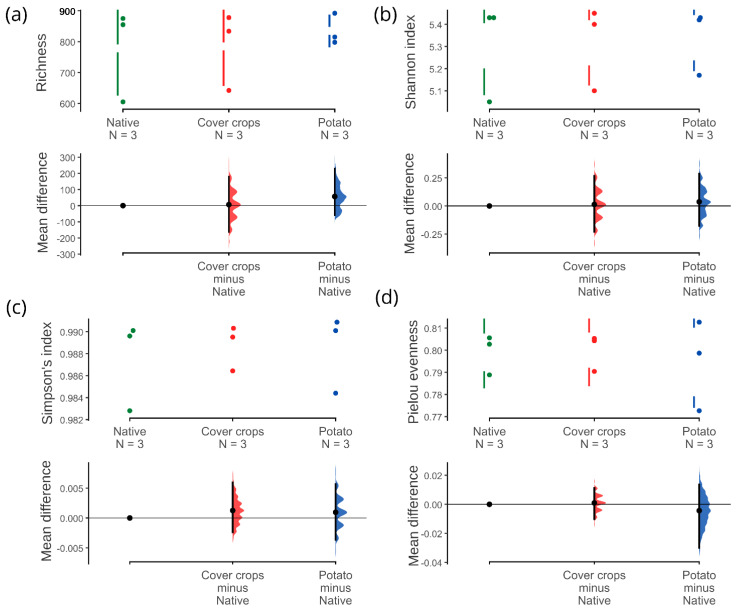
Gardner–Altman plots representing values of the alpha diversity metrics of the soil bacterial community in areas of cover crop (CC), potato crop (PC) and native Cerrado (NC) (Cerrado sensu stricto). (**a**) Richness, (**b**) Shannon index, (**c**) Simpson’s index, and (**d**) Pielou evenness. The observed variations for the alpha diversity metrics of the soil bacterial community were analyzed between the different areas. The plots present the effect strengths (Hedge’s g) and their 95% confidence intervals for nutrients and cations where the confidence intervals do not include the value 0, indicating significant differences. In the Gardner–Altman plots, the distribution of sampled values is displayed at the top, while Hedge’s g is shown at the bottom as a dot, accompanied by a horizontal line on the axis. The vertical error bars represent the 95% bootstrap confidence intervals, based on 5000 bootstrap samples, together with the distribution of the resamplings.

The alpha diversity of the soil bacterial community among NC, CC, and PC areas was evaluated by the indices of species richness (S), Shannon diversity (H’), Simpson diversity, and Pielou evenness. (J) Appendix A (Figure A5). Interestingly, we did not find significant differences for any of the alpha diversity metrics among the three areas. The highest values of S, H’, and 1-Simpson were observed in the PC area (835 ± 40.89, 5.34 ± 0.01, and 0.988 + 0.002, respectively). The highest J value was observed in the NC area (0.799 ± 0.007).

**Figure 5:**
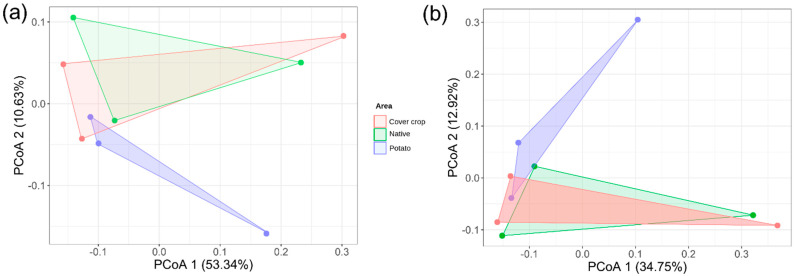
Analysis of principal coordinates (PCoA) based on the Bray–Curtis and Jaccard distance matrices of the soil bacterial community among cover crop, native Cerrado, and potato cultivation areas. In (**a**) PCoA based on the Bray–Curtis distance matrix; in (**b**) PCoA based on the Jaccard distance matrix. The percentage of variation explained by the plotted main coordinates is indicated on the *X* and *Y* axes. Colored polygons indicate the clustering of bacterial communities between areas.

The beta diversity of the soil bacterial community among the three areas was assessed at the ASV level to compare similarities and dissimilarities. For this, we use Bray–Curtis and Jaccard distance matrices. The Principal Coordinates analysis (PCoA) based on the Bray–Curtis and Jaccard distance matrices demonstrated overlap of the CC area with the NC area and separation of the PC area (Figure 5a,b). Using the Bray–Curtis matrix, a total of 63.97% of the variation in the soil bacterial community among areas was explained by the X (53.34%) and Y (10.63%) axis of the PCoA. For the Jaccard distance matrix, a total of 47.67% of the variations in the soil bacterial community among areas were explained by the X (34.75%) and Y (12.92) axes of the PCoA. The PERMANOVA analysis of variance based on the Bray–Curtis matrix showed that Zn (R^2^ = 0.29, *p* = 0.041) was significant in the composition of the soil bacterial community among areas (Table 1). PERMANOVA analysis based on the Jaccard distance matrix showed that Al (R^2^ = 0.22, *p* = 0.03) had an effect on the community.

**Figure 6:**
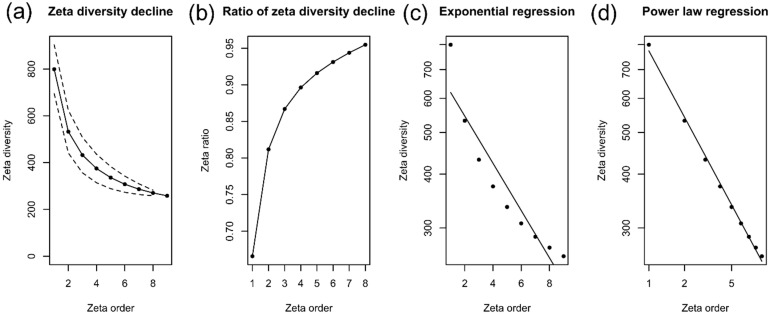
Decline in zeta diversity and model adjustment of the soil bacterial community in cover crop, native Cerrado, and potato crop areas. (**a**) zeta diversity decline; (**b**) 0 to 1 scaled proportion of zeta diversity decline, represented shared ASVs and ASV retention rate through ζ i, respectively. (**c**) zeta diversity model suitable for exponential regression and (**d**) zeta diversity model suitable for power law. The exponential regression fit suggests equal and stochastic turnover among ASVS. Fitting to power law regression indicates turnover driven by ecological niche differentiation processes and rare ASVs with low probabilities of being found in the data set.

The analysis of zeta diversity (ζ) with a sample selection scheme of all combinations not structured geographically allowed the evaluation of the trends in the turnover of the soil bacteria community among the three areas (Figure 6). The decline in zeta diversity (average number of shared ASVs) with zeta order (number of sampling units) was slightly accentuated (Figure 6a). This means that the number of shared ASVs decreased markedly as more sampling units were included for the zeta diversity estimation, with an average sharing of 258 ASVs among all samples (zeta order = 9). Furthermore, the sharp decline suggests that the composition change is mainly due to rare ASVs. The retention rate showed an upward curve, demonstrating that common, but not rare, ASVs are being retained in higher zeta orders (Figure 6b). This, therefore, indicates the scale at which ASVs can be considered common and rare. The zeta decline fits better to a power law regression (Akaikes information criterion (AIC) = −55.16) than the exponential regression (Akaikes information criterion (AIC) = −21.58), suggesting that the ASVs are distributed deterministically among the three areas (Figure 6c,d). Furthermore, the best fit to the power law model suggests a turnover driven by ecological niche differentiation processes and a low probability of finding rare ASVs.

### 3.4. ASVs Co-Occurrence and Sharing Networks

The ASV sharing network demonstrated that 832 ASVs were present in the three areas (Appendix A—Figure A6). The CC area had 200 unique ASVs, the NC had 163 unique ASVs, and the PC had 205 unique ASVs (Appendix A—Figure A6). Regarding ASV sharing, the CC area shared 78.11% of its ASVs (953 ASVs) with the NC area and 74.52% of ASVs (939 ASVs) with the PC area. The potato area shared 75.00% of its ASVs (936 ASVs) with the NC area.

We used co-occurrence network analysis, based on SparCC correlation values, to explore the co-occurrence patterns of the bacterial community among the three areas (Appendix A—Figure A7). Overall, the network had 619 nodes (619 ASVs), 14,762 edges (interactions), a density of 0.039, a clustering coefficient of 0.185, and avg. number of 47,696 neighbors were identified. The majority of nodes present in the network belong to Proteobacteria (23.34%), followed by Actinobacteriota (15.54%), Firmicutes (14.52%), Bacteroidota (9.38%), Chloroflexi (8.94%), Acidobacteriota (8.65%), Verrucomicrobiota (5.43%), and other phyla (abundance < 5%). The positive–negative relationship found on the network was relatively balanced (53.35%: 46.65%). The MCODE analysis demonstrated that in the co-current network, there are 26 densely connected clusters. The four clusters with the highest MCODE values are shown in Appendix A—Figure A7b–e. The ASVS ASV_1094 (Ktedonobacteraceae) and ASV_1632 (Ktedonobacterales), both belonging to Chloroflexi, presented the highest degree values and highest betweenness (a measure of centrality in a graph based on the shortest paths).

### 3.5. Habitat Preference and Functional Capacity of the Soil Bacterial Community

**Figure 7:**
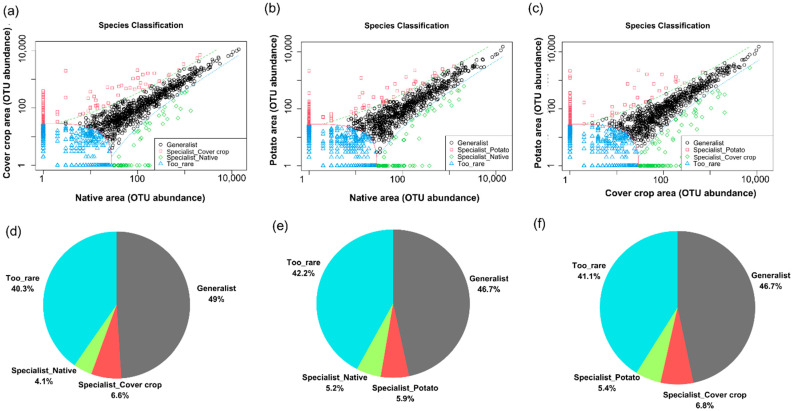
Multinomial species classification method (CLAMtest) categorizing soil bacterial ASV among cover crop, native, and potato cultivation areas. The ASVs were classified into four categories: specialists in area x (cover crop, native Cerrado, and potato crop), generalists, too rare to be classified, and specialists in y (cover crop, native Cerrado, and potato crop). (**a**) cover crop area x native area; (**b**) potato area x native area; (**c**) potato area x cover crop area; (**d**) native area x cover crop area; (**e**) native area x Potato area; (**f**) potato area x cover crop area.

To quantify the differences among the soil bacterial community among the three areas, we applied CLAMtest, a multinomial species classification method. CLAMtest classified bacterial ASVs into four categories: specialists in area x (cover crop, native Cerrado, potato cultivation), generalists, too rare to be classified, and specialists in area y (cover crop, native Cerrado, potato cultivation). In general, our results show that the bacterial community among the areas is basically composed of generalist and/or rare ASVs with low levels of specialization (Figure 7). When the NC area was compared with the CC area, 40.3% of ASVs were classified as rare, 49% as generalists, 4.1% as NC area specialists, and 6.6% as CC area specialists. When the NC area was compared with the PC area, 42.2% of the ASVs were rare, 46.7% were generalists, 5.2% were NC area specialists, and 5.9% were PC area specialists. When the PC area was compared with the CC area, 41.1% of the ASVs were classified as rare, 46.7% as generalists, 5.4% as PC area specialists, and 6.8% as CC area specialists.

The “Global Mapper” module in iVikodak classified the bacterial communities from the three sites (cover crop, native, and potato) into ten distinct functional profiles. These profiles were associated with metabolic processes such as carbohydrate metabolism, amino acid metabolism, vitamin and cofactor biosynthesis, energy metabolism, and DNA replication and repair. The functional profiles were visualized in two categories: (1) higher-level or specific metabolic pathways linked to central metabolism, including purine and pyrimidine metabolism, pyruvate metabolism, and amino sugar metabolism (Appendix A—Figure A8a); and (2) complementary metabolic categories related to broader energy metabolism, DNA replication and repair, and cofactor and vitamin metabolism (Appendix A—Figure A8b). Interestingly, bacterial communities in the NC (native) site exhibited the highest functional activity across all profiles. Functional analysis using the FAPROTAX database revealed distinct metabolic profiles linked to key biogeochemical processes, such as carbon cycling (C cycle), nitrogen cycling (N cycle), sulfur cycling (S cycle), energy metabolism, and other functional categories (Appendix A—Figure A9). Metabolic profiles were categorized into distinct metabolic pathways, emphasizing the metabolic capabilities of the bacterial community at each site (Appendix A—Figure A10). In the carbon cycle, fermentation was the predominant microbial activity across all three areas. In the nitrogen cycle, nitrate reduction and nitrogen fixation were the most abundant processes. In the sulfur cycle, sulfate respiration and sulfur compound respiration were the dominant microbial activities, with higher values observed in cover crop and potato cultivation areas. Dark oxidation of sulfur compounds exhibited low values with minimal variation among the areas. The predominant functional groups identified were aerobic chemoheterotrophy and anaerobic chemoheterotrophy (Appendix A—Figure A11).

## 4. Discussion

### 4.1. Physicochemical Properties and Soil Enzymes

A variety of environmental factors are important indicators for evaluating soil quality [44]. In our study, we found significant differences in all the physicochemical parameters of the soil that we analyzed. As expected, soils from the NC area showed the highest Al values in contrast to the CC and PC areas. This is due to the fact that naturally, the Cerrado soils are acidic and have high concentrations of aluminum [45]. The soil from the PC area presented the lowest OM and C values compared to the NC area. Potato cultivation occurs at the expense of conventional soil preparation, which results in major disturbances in its physicochemical properties, which can alter carbon sequestration and organic matter levels [46,47,48,49]. In contrast, the increase in OM and C levels in the CC area may be the result of organic carbon sequestration, as it has been shown that this type of management increases soil carbon levels [11].

High concentrations of Zn were found in the PC and CC areas, being 2.5× higher in the PC area and 6× higher in the CC area compared to the NC area. This increase in Zn concentrations in Cerrado soils is due to intense agricultural practices (excessive use of fertilizers, agrochemicals, etc.) [50]. Furthermore, the soils in the CC and PC areas showed high CEC values, which may indicate a soil salinization process [51,52,53]. These two characteristics are indicative of the soil degradation process [50,51].

The arylsulfatase and phosphatase enzymes showed higher activity values in the NC area. Furthermore, the activity of these enzymes was negatively correlated with high concentrations of Ca, Mg, Zn, pH (>6.0), and carbon. Therefore, the reduction in the activity of these enzymes in areas of CC and PC may be related to the addition of chemical fertilizers or low levels of OM present in these areas. Furthermore, the high concentrations of Zn in these areas may be one of the limiting factors for the low activity of these enzymes since Zn can act as an inhibitor of enzymatic activity [54]. On the contrary, the enzyme β-Glucosidase showed higher activity values in the CC and PC areas, being positively related to Ca, Mg, Zn, and pH (>6). This greater activity of β-Glucosidase in these two areas may be related to the quality and quantity of plant residues present in these areas, changes in pH to ideal conditions for enzyme activity, increase in cofactors, etc. [21,22].

### 4.2. Community of Soil Bacteria

As microbial community structure is generally related to soil properties, we further investigated the soil bacterial community composition among the three areas. The three bacterial phyla with the highest relative abundance in the three areas were Proteobacteria, Actinobacteriota, and Acidobacteriota. However, the relative abundance of this phylum did not differ significantly, suggesting that the regenerative agriculture practices systems evaluated here may not have a significant effect on the composition of soil bacteria at the phylum level. This lack of clear response to Cerrado soil bacteria under the crop–livestock integration system and native forest has already been documented [55]. These three phyla exert numerous critical ecological functions in the soil and are commonly reported as dominant groups [56,57,58,59,60]. The Proteobacteria phylum has high metabolic plasticity and may be involved in the cycling of carbon, nitrogen, sulfur, and plant growth promotion [56,59,60]. The phyla Actinobacteriota and Acidobacteriota can act in the OM degradation process, which helps to maintain high soil carbon levels [57,58,61,62,63].

At the taxonomic class level, small variations were observed between areas, while at the ASV level, these variations were more pronounced. However, we did not find significant differences. These results can be explained by numerous factors. Firstly, it is known that the taxonomic structure of the soil bacteria community is generally rich and diverse, composed of a few abundant groups and many rare groups with low abundance [64,65,66,67,68], which is in line with our results. These stable taxonomic distribution patterns of the soil bacteria community can be explained by numerous factors, which include adaptive capabilities [57,58,61,62,63]. Furthermore, it is believed that bacteria are capable of occurring in wide contrasting biogeographical ranges, entering a state of dormancy, which leads to limited turnover [69,70]. Other hypotheses should also be considered, such as insufficient sampling effort [71], low taxonomic resolution resulting in the exclusion of rare ASVs [72,73], low sequencing depth, or rarefied data [74]. Therefore, numerous factors must be considered to explain the similarities in the taxonomic composition of the bacterial community among the CC, NC, and PC areas.

In both cultivation areas, regenerative agriculture practices are implemented. Such practices have been shown to be effective in improving bacterial community structure, resulting in healthier soil and agriculture [75]. Furthermore, there are studies that indicate that food generated through regenerative agriculture has superior nutritional quality, as these techniques, in addition to promoting greater microbial diversity in the soil, also favor the microbiota of plants and animals, including humans [76].

The alpha diversity indices showed that the bacterial communities among the areas are rich and diverse. However, no significant differences were found. Results similar to those found in our study have already been found in other studies that evaluated the alpha diversity of soil bacterial communities between different treatments [57,58,59,67,68,77]. These works demonstrate that cultivation practices or types of cultivated plants affect the physicochemical characteristics of the soil. However, the richness, species diversity, and evenness of the bacterial community between treatments remain stable, being composed of a few abundant groups, which mainly belong to the phyla Proteobacteria, Acidobacteriota, Actinobacteriota, and Firmicutes. This is due to the fact that species belonging to these taxonomic groups have high functional plasticity and can occur in different biogeographic and environmental gradients as dominant groups [57,58,59,67,68,77].

The sharing and co-occurrence networks demonstrated that 47.51% of the ASVs (832 ASVs) were common to the three areas (Appendix A—Figure A7). Furthermore, CLAMtest and the network demonstrated that the bacterial community among the three areas was mainly composed of generalist and rare ASVs. These corroborating findings are supported by analyses of alpha diversity and relative abundance and are in agreement with other studies, demonstrating that the taxonomic structure of the soil bacteria community is composed of generalist and rare species [57,58,64,65,66].

Bray–Curtis and Jaccard distance-based PCoA demonstrated an overlap of bacterial communities present in the CC area with the NC area and separation from the PC area. The dissimilarities between areas were driven by a few ASVs, which individually contributed ~1% of the total dissimilarity. The PERMANOVA analysis showed that Al and Zn were the main soil characteristics that helped explain the dissimilarities among areas (*p* < 0.05). Zn presented higher concentrations in areas of CC and PC. Al presented the highest concentrations in the NC area, which is a characteristic of Cerrado soils [78]. Zn has already been correlated with changes in the composition of microbial communities [59]. Zinc is a micro-nutrient that acts as a cofactor for numerous microbial enzymes. Some studies have shown that a high level of zinc in the soil has a strong effect on bacterial communities, as it can directly affect the metabolism of these microorganisms [54,79,80].

The analysis of zeta diversity (ζ) revealed an intriguing pattern in the distribution of ASVs across the three studied areas. The decline in zeta diversity as the zeta order increases, along with a reduction in the number of shared ASVs, suggests that the bacterial community composition is driven by the presence of rare ASVs, which become more apparent as additional sampling units are incorporated into the analysis. This pronounced decline indicates a deterministic distribution of ASVs. The retention curve pattern, showing the persistence of common ASVs at higher zeta orders, further supports the idea that rare ASVs are less prevalent at larger sampling scales. This finding is particularly significant, as rare species are often underrepresented or overlooked in diversity analyses, leading to an incomplete understanding of community dynamics. These results, together with beta diversity data, can help to understand the mechanisms of structuring the soil bacterial community.

The in silico prediction demonstrated that the NC area presented the highest values of functional diversity of the soil bacterial community compared to the others. This was also observed in the enzymatic activity, which supports the results obtained from the in silico prediction. The greater functional diversity observed in the NC area may be related to the higher levels of OM [81]. The lower values of functional diversity observed in cultivated areas may be due to low levels of OM and high levels of CEC and Zn, which impact the metabolism and numerous enzymatic activities of these microorganisms [52,53,54,79,80].

## 5. Conclusions

The data obtained indicated significant impacts on soil physicochemical properties and enzymatic activity, which directly reflect the dynamics of bacterial communities. These results provide valuable information on conservation, soil carbon loss, and soil health, serving as indicators of soil quality in the Cerrado under different forms of land use. The increase in concentrations of metals, such as Zn, in cultivated areas, coupled with the reduction of soil organic matter and carbon, suggests that the agricultural practices evaluated may lead to soil degradation in the Cerrado, compromising ecosystem functionality. In addition, enzymatic activities, such as arylsulfatase and phosphatase, showed negative correlations with high levels of Ca, Mg, Zn, and pH, evidencing the relationship between the addition of fertilizers and the liming process with the decrease in the efficiency of these enzymes, which are essential for nutrient cycling and soil health. While small variations were observed in bacterial communities at the taxonomic class level across different land use systems (cover crops, potato cultivation, and native Cerrado), more pronounced variations were recorded at the amplicon sequence variant (ASV) level.

On the other hand, the regenerative management system demonstrated promising potential to mitigate the negative impacts of intensive agricultural practices. Although no significant differences in alpha diversity between treatments were identified, the analysis of co-occurrence networks and beta diversity revealed a clear influence of soil characteristics, such as Al and Zn levels, on bacterial community structure. These findings suggest that maintaining both taxonomic and functional diversity in regenerative management areas while preserving optimal soil conditions may be crucial for promoting long-term soil sustainability and health.

## Figures and Tables

**Table 1:** Adonis2 PERMANOVA analysis showing the soil parameters correlation with soil bacterial communities among cover crop, native Cerrado, and potato cultivation areas.

Distance Method	Variable	R^2^	*p* Value
	Area	0.16350	0.678
	Zn	0.29698	0.041
Bray–Curtis	OM	0.07801	0.601
	Residual	0.46152	NA
	Total	1	NA
	Area	0.20946	0.604
	Al	0.22323	0.033
Jaccard	Carbon	0.09898	0.568
	Residual	0.46833	NA
	Total	1	NA

Al: aluminum; Zn: zinc; OM: organic matter. NA = Not applicable.

**Figure A6:**
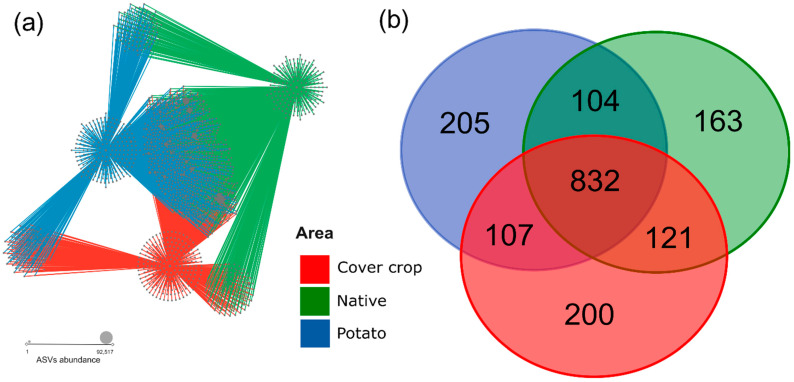
Network and venn diagram of sharing ASVs of the soil bacterial community among cover crop, native Cerrado, and potato cultivation areas. In (**a**), structure of the ASV sharing network of the soil bacterial community among cover crop, native Cerrado, and potato cultivation areas. (**b**) Venn diagram showing the number of ASVs shared among cover crop, native Cerrado, and potato cultivation areas.

**Figure A7:**
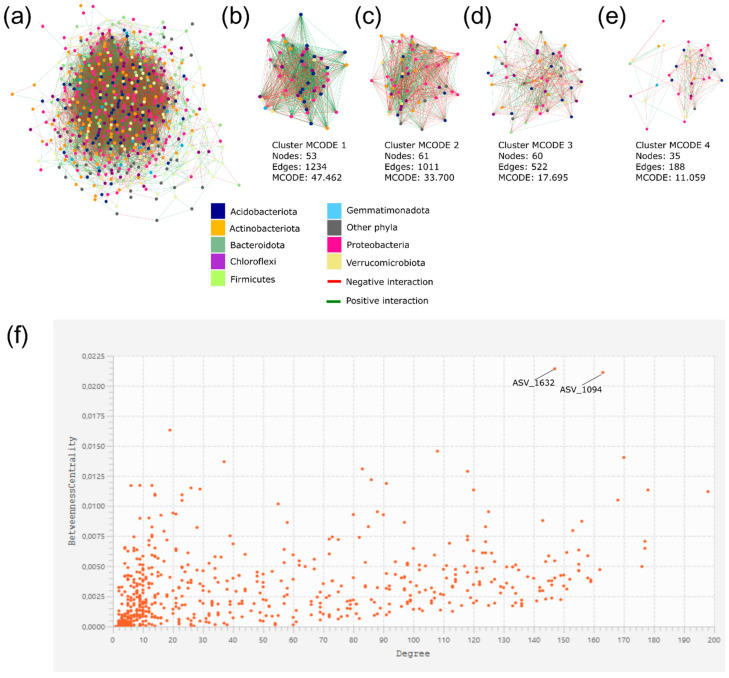
SparCC correlation networks and MCODE interaction networks (densely connected network components) of the soil bacterial community present in cover crop, native Cerrado and potato cultivation areas. (**a**) SparCC correlation network created from a matrix of filtered ASVs to remove ASVs with values <100 reads and ASVs that were not present in >50% of the samples. (**b**–**e**) MCODE interaction networks showing the main most connected groups in the SparCC network. The color of the nodes corresponds to the different bacterial phyla. Only significant correlations (*p* ≤ 0.001 based on 100 bootstrap replicates) with absolute correlation magnitude ≥ 0.6 are shown. (**f**) Betweenness, centrality, and degree of each ASV in the SparCC correlation network. Nodes with high betweenness centrality and high degree values are considered key taxa in networks. Figure created using CYTOSCAPE version 3.9.1.

**Figure A8:**
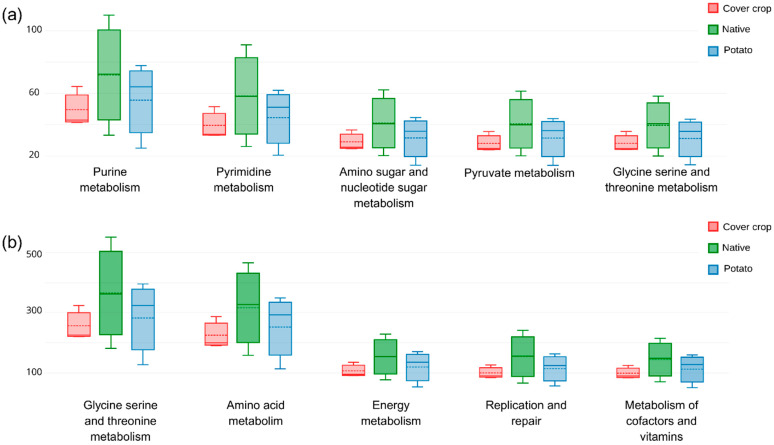
Functional profiles of the soil bacterial community in cover crop, native Cerrado, and potato cultivation using the “Global mapper” tool in iVikodak. (**a**) Higher-level or specific metabolic pathways related to central metabolism. (**b**) Complementary metabolic categories, including energy, cellular repair, and cofactors.

**Figure A9:**
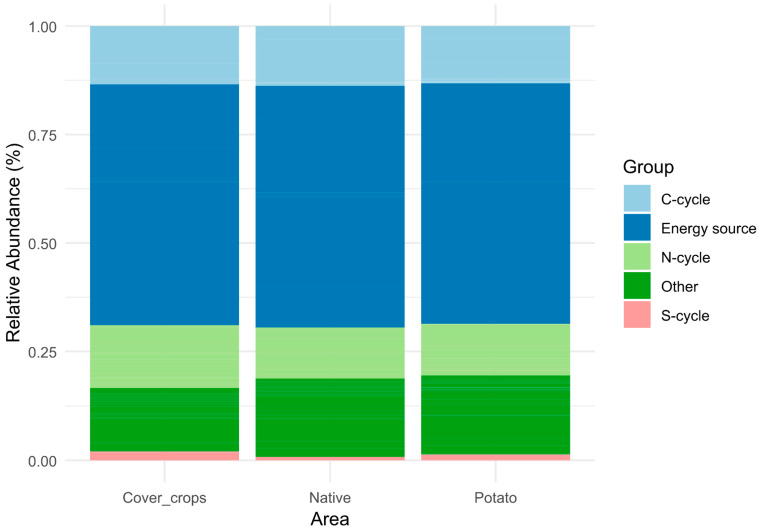
Functional profiles of the soil bacterial community in cover crop, native Cerrado, and potato cultivation using the FAPROTAX.

**Figure A10:**
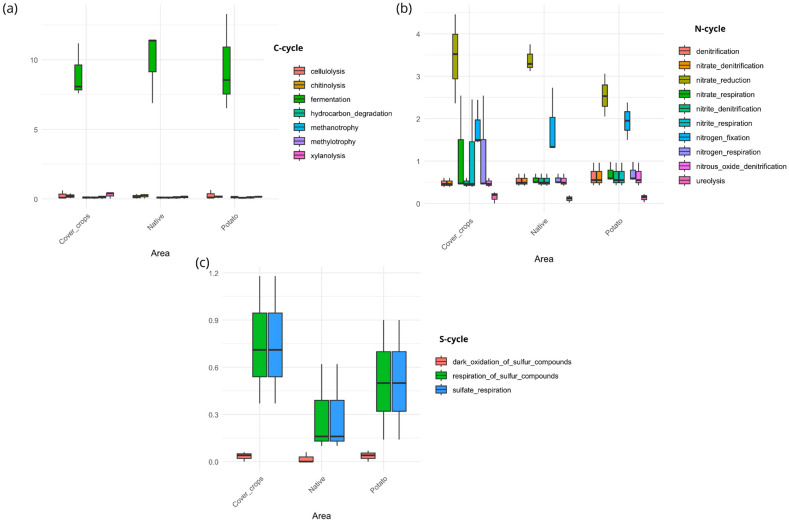
Functional profiles of the soil bacterial community in cover crop, native Cerrado, and potato cultivation using the FAPROTAX. (**a**) processes related to the carbon cycle; (**b**) processes related to the nitrogen cycle; (**c**) processes related to the sulfur cycle.

**Figure A11:**
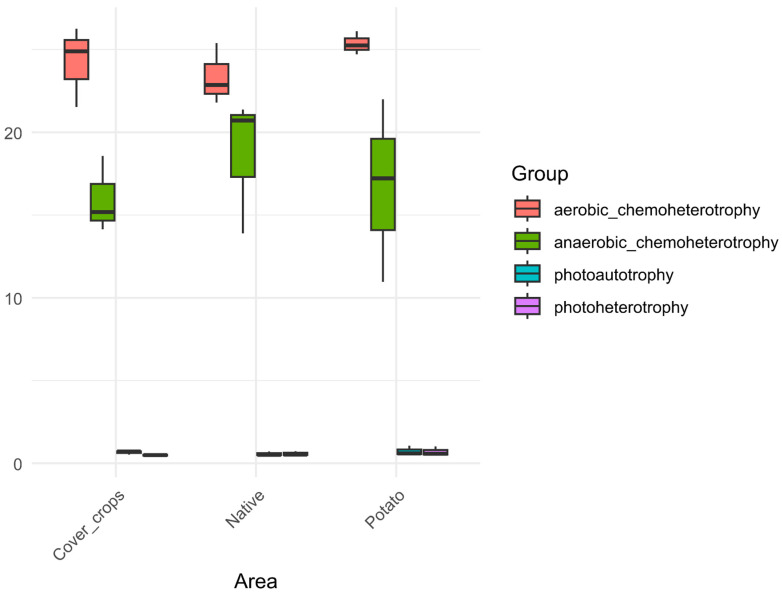
Energy source profiles of the soil bacterial community in cover crops, native Cerrado, and potato crops, as determined using FAPROTAX (version 1.2.9).

## Data Availability

The original contributions presented in this study are included in the article. Further inquiries can be directed to the corresponding author.
